# 

**Published:** 1866-10

**Authors:** 


### CONTENTS.

### ORIGINAL CONTRIBUTIONS.

ART. XXXV. EPIDEMIC CHOLERA.—A FEW REMARKS ON ITS PATHOLOGY AND TREATMENT. By R. DEXTER, M.D., Chicago, 577
ART. XXXVI. OBSERVATIONS ON SCURVY, AND ITS CAUSES AMONG THE U. S. COLORED TROOPS OF THE 25TH ARMY CORPS, DURING THE SPRING AND SUMMER OF 1865. By S. HEMENWAY, M.D., late Surgeon 41st U.S. Colored Troops, 582
ART. XXXVII. TAPE-WORM.—(*Tœnia Solium.*) By J. V. HERRIOTT, M.D., Valparaiso, Ind., 587
ART. XXXVIII. REPORT ON CEREBRO-SPINAL MENINGITIS. By J. S. JEWELL, M.D., Prof. Anatomy, Chicago Medical College; Member of Illinois State Medical Society, etc. (Read to the Illinois State Medical Society, June, 1866,) 589

### BOOK NOTICES.

A Guide to the Practical Study of Diseases of the Eye; with an Outline of their Medical and Operative Treatment. By James Dixon, F.R.C.S., Surgeon to the Royal Ophthalmic Hospital, Moorfields, London, 627
Instructions in the Preparation, Administration, and Properties of Nitrous Oxide, Protoxide of Nitrogen, or Laughing Gas. By Geo. T. Barker, D.D.S., Prof. of Principles of Dental Surgery and Therapeutics, in the Pennsylvania College of Dental Surgery, etc., 627
The Physician's Visiting List for 1867, 628
Manual of Materia Medica and Therapeutics: Being an Abridgement of the late Dr. Pereira's Elements of Materia Medica, arranged in conformity with the British Pharmacopœia. By Frederic John Farre, M.D., Cantab., etc., assisted by Robert Bentley, M.R.C.S., etc., etc., Robert Warington, F.R.S., etc., and Horatio C. Wood, Jr., M.D., etc. 628

### EDITORIAL.

Clinical Instruction in Chicago, 629
Mortality in Chicago for the Month of August, 630
Report of the Committee on the Sanitary Condition of the City, and the Prevalence of Diseases, during the Month of July, 1866, 631
The Baneful Effects of Nicotine Prevented, 588
The Cholera Cases in Chicago during the Month of September, 1866, 636
Chicago Medical College, 637
Disinfection and Deodorization, 637

